# The impact of creative anxiety on professional identity among master’s nursing students: a chain mediation effect of psychological resilience and achievement motivation

**DOI:** 10.1186/s12912-025-03535-6

**Published:** 2025-07-07

**Authors:** Yao Ding, Xiaolan Guo, Ruifeng Wang, Lu Xu, Shajie Hou, Fengjiao Chang

**Affiliations:** 1 School of Nursing, Shaanxi University of Chinese Medicine, Xianyang, Shaanxi 712046 China; 2 Department of Nursing, The Affiliated Hospital of Xizang Minzu University, Xianyang, Shaanxi 712046 China; 3 Department of Nursing, The Second Affiliated Hospital of Shaanxi University of Chinese Medicine, Xianyang, Shaanxi 712046 China

**Keywords:** Master's nursing students, Creative anxiety, Professional identity, Psychological resilience, Achievement motivation, Chain mediation effect

## Abstract

**Objective:**

To determine whether psychological resilience and achievement motivation are significant mediators in the relationship between creative anxiety and professional identity among master’s nursing students.

**Methods:**

A questionnaire survey was conducted with 366 master’s nursing students from four universities in Shaanxi Province. The survey used the General Information Questionnaire, Creative Anxiety Scale, Professional Identity Scale, Psychological Resilience Scale, and Achievement Motivation Scale. Pearson’s r was used to examine the relationships among the variables, and a structural equation model (SEM) was employed to clarify the mediating effects.

**Results:**

In this study, the mean scores of creative anxiety, professional identity, psychological resilience, and achievement motivation among 366 master’s nursing students were (12.92 ± 3.58), (48.47 ± 12.76), (28.32 ± 7.71), and (6.23 ± 1.83), respectively. Creative anxiety showed significant negative correlations with professional identity (*r* = − 0.668, *P* < 0.001), psychological resilience (*r* = − 0.537, *P* < 0.001), and achievement motivation (*r* = − 0.503, *P* < 0.001). Conversely, professional identity demonstrated positive correlations with both psychological resilience (*r* = 0.713, *P* < 0.001) and achievement motivation (*r* = 0.663, *P* < 0.001). Mediation analysis revealed that the total effect of creative anxiety on professional identity (β = −0.458) comprised a direct effect (β = −0.124) and an indirect effect mediated through psychological resilience and achievement motivation (β = −0.334). A significant serial mediation effect was observed (β = −0.092), with psychological resilience and achievement motivation serving as sequential mediators between creative anxiety and professional identity. This serial mediation accounted for 8.97% of the total effect, with a 95% confidence interval of (− 0.211 ~ − 0.009).

**Conclusion:**

There is a multiple mediation effect among creative anxiety, professional identity, psychological resilience, and achievement motivation among master’s nursing students. Creative anxiety is closely related to professional identity. Therefore, it is recommended that university administrators focus on enhancing the psychological resilience and achievement motivation of master’s nursing students to reduce their creative anxiety and improve their professional identity.

**Clinical trial number:**

Not applicable.

## Introduction

Professional identity in nursing is a dynamic and evolving concept that is essential for both individual nurses and the nursing profession as a whole. It is shaped by continuous experiences and practice, as well as an individual’s degree of recognition and acceptance of the nursing profession [1]. Nurses with a strong professional identity may experience less role stress and turnover, be more motivated and engaged in their work, and be able to provide higher quality care [2, 3]. Professional identity formation starts in the training phase of students’ education. Research shows mixed findings regarding the professional identity of nursing students; However, a lower level of professional identity among master’s nursing students was consistently observed [4]. This phenomenon may be attributed to the dual pressures they face: heavy academic research demands and the challenging process of translating theoretical knowledge into practical skills [5]. Diminishing professional identity will ultimately decrease the quality of care and motivation for professional learning of nursing students. This is particularly significant since highly educated nurses have been shown to reduce patients’ mortality rates, shorten hospital stays, and lower health care costs [6, 7]. Furthermore, within rapidly evolving healthcare systems, professional identity formation among master’s nursing students represents a critical leadership cohort in the discipline. This development holds dual significance for both individual career trajectories and the advancement of nursing’s professionalization and scholarly progress, while simultaneously serving as a crucial determinant of workforce retention.

Multiple determinants influence professional identity development among nursing students, including demographic characteristics such as gender (with females reporting higher levels) and educational level (with baccalaureate students demonstrating stronger identity than master’s students in the reported findings) [8]. Additionally, both positive and negative psychological factors (e.g., negative emotions [9], resilience [10]) have well-established significant correlations with professional identity formation. However, the relative contributions of these psychological factors remain unclear and warrant further empirical investigation.

To understand the psychological dynamics involved, Conservation of Resources (COR) theory provides a relevant foundational framework. This theory of stress and motivation posits that individuals strive to minimize resource losses while maximizing gains and has emerged as key to understand employee well-being and future job design [11]. Notably, it elucidates how individuals safeguard psychological assets to prevent burnout [12]. For master’s nursing students, resource depletion constitutes the primary antecedent of psychological stress and negative emotions like anxiety [13]. To mitigate such losses, individuals mobilize protective psychological resources such as resilience and motivation [14]. Adequate resource conservation prevents professional identity erosion, burnout, and diminished professional accomplishment. Nevertheless, empirical research has yet to examine the mediating mechanisms through which nurses’ resource loss impacts professional identity formation among this master’s student population.

Growing research examines the link between creative engagement and professional identity development in nursing students. For instance, a recent study has shown that there is a positive correlation between creative ability and professional identity, with students who have a stronger sense of creative ability demonstrating higher levels of professional identity [15]. However, a potential barrier to the fulfillment of creative ability that has recently been identified is creative anxiety [16]. This anxiety occurs when individuals face situations requiring creative thinking and produces higher anxiety ratings than comparable non-creative situations [17]. This challenge is pertinent for master’s nursing students, who are being prepared for roles as advanced practice nurses, where innovation is an inherent professional expectation [18]. When students avoid experiencing this heightened anxiety and fail to develop these real-world creative pursuits, a decline in perceived professional value occurs, which consequently undermines their professional identity [19]. Meanwhile, negative emotions, such as anxiety and high stress, are known risk factors for professional identity of nursing students [20]. Although the relationship between creative anxiety and professional identity have been noticed, the underlying mechanism remains insufficiently unexplored.

Research suggests that positive psychological traits, which are intrinsic resources that can be developed and enhanced, play a key role. Emphasizing the cultivation and management of psychological strengths can help individuals better recognize and discover their potential, thereby neutralizing the influence of creative anxiety [21]. Among these traits, psychological resilience and achievement motivation, as key components, play a crucial role in helping individuals cope with challenges and attain goals [22]. Psychological resilience refers to an individual’s intrinsic ability to demonstrate positive adaptation and cope with environmental challenges when faced with stressful situations [23]. Given that nursing master’s students are advanced professionals facing high standards across competence, research, teaching, and practice, possessing strong resilience and adaptability is particularly crucial. Importantly, psychological resilience is a significant factor influencing professional identity formation [24]. Individuals with higher resilience possess the ability to self-regulate and effectively manage psychological stress, which helps reduce the occurrence of negative emotions (e.g., depression and anxiety). In clinical practice, such individuals can clearly recognize their professional goals and values, which further strengthens their sense of professional identity [23].

Achievement motivation is another intrinsic driving force that enables individuals to persistently and proactively accomplish tasks in pursuit of expected goals, encompassing both motivation for success and to avoid failure [25]. Empirical evidence highlights it as a critical neurocognitive driver in sustaining master’s nursing students’ perseverance through research-related adversities. Through its regulatory effect, achievement motivation influences professional identity—stronger achievement motivation predicts higher professional identity levels and can serve as a predictive factor for it [26].

In summary, while creative anxiety, psychological resilience, and achievement motivation can influence professional identity, the underlying mechanism among those factors remains insufficiently explored. To construct the argument of how creative anxiety affects nursing master’s students’ professional identity, we draw upon the COR theory. Resource acquisition and maintenance serve as protective mechanisms against the threat of depletion, with demonstrable effects on both psychological recovery and personal growth outcomes [27]. Within this framework, creative anxiety, as a threat to psychological resources, may persistently consume individuals’ cognitive and emotional resources, inadequately replenishing resources effectively, thereby establishing a direct link to subsequent professional identity diminishment. In this proposed mechanism, psychological resilience serves as a critical resource that helps protect and maintain existing psychological resources, counteracting depletion caused by creative anxiety, and thus preserving resources to prevent professional identity erosion. Achievement motivation functions as a driver of resource gain that positively predicts professional identity and facilitates resource accumulation (e.g., enhanced professional identity) [28]. As important antecedent variables, psychological resilience has been proved positively predict achievement motivation [29]. Based on this integrated theoretical framework, we hypothesize that psychological resilience and achievement motivation may play a chain-mediating role between creative anxiety and professional identity among nursing master’s students.

**Fig. 1:**
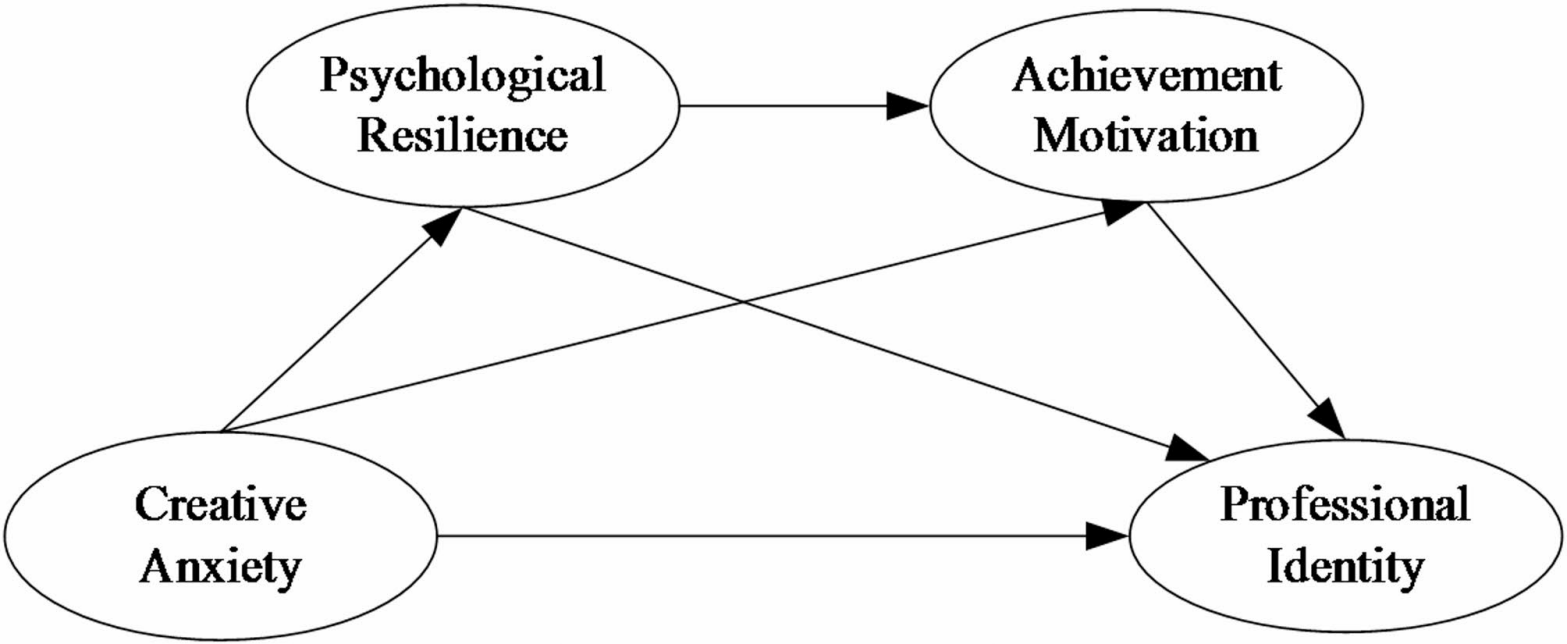
Conceptual model

This study proposes four hypotheses (Fig. 1):

### H1

Creative anxiety negatively affects the professional identity of master’s nursing students.

### H2

Psychological resilience mediates the relationship between creative anxiety and professional identity among master’s nursing students.

### H3

Achievement motivation mediates the relationship between creative anxiety and professional identity among master’s nursing students.

### H4

Psychological resilience and achievement motivation sequentially mediate the relationship between creative anxiety and professional identity among master’s nursing students.

## Methods

### Study design and participants

From April to July 2023, a convenience sampling method was used to select master’s nursing students from four universities in Shaanxi Province who met the inclusion criteria to participate in the study. The inclusion criteria were: (1) Full-time professional degree master’s nursing students enrolled from 2021 to 2023 and (2) informed consent and voluntary participation in the study. Exclusion criteria: (1) part-time graduate students, (2) graduate students on leave of absence for various reasons, and (3) graduate students pursuing an academic degree in nursing.

The sample size calculation for this study adhered to the guidelines proposed by Kline [30]. These guidelines recommend that the sample size should be 10–20 times the number of research variables. Since this study involved 24 variables and accounted for a 15% rate of invalid questionnaires, the required sample size was estimated to be between 283 and 565 participants. Ultimately, the study surveyed 366 master’s nursing students.

### Research instruments

#### General information questionnaire

The researchers designed this questionnaire, which included the following items: age, gender, marital status, grade level, area of specialization, research achievements to date, average weekly time spent studying independently, study efficiency, future career goals, level of communication within the team, intensity of supervisor guidance, and graduation requirements for the master’s degree.

#### Creativity anxiety scale (CAS)

The Chinese version of the Creativity Anxiety Scale (CAS), developed by Zhiting Ren [31], was used. The CAS contains two dimensions, creative and non-creative anxiety, with a total of 12 items. Respondents rate each item on a 5-point Likert scale ranging from “not at all” to “very much,” and scores are assigned from 0 to 4.

The scores for each dimension range from 0 to 24, with higher scores indicating higher levels of anxiety in that dimension. This scale has good structural validity, and the Cronbach’s alpha coefficient is 0.90. In this study, the Cronbach’s alpha coefficient for this scale was 0.930.

#### Professional identity scale for nursing students (PISNS)

The Professional Identity Scale for Nursing Students (PISNS), developed by Yufang Hao [32], was used. The scale includes five dimensions: professional self-concept; job retention, benefits, and turnover risks; social comparison and self-reflection; autonomy in career choice; and social persuasion. There are a total of 17 items on the scale. Respondents are asked to rate each item on a 5-point Likert scale ranging from 1 (“very inconsistent”) to 5 (“very consistent”), resulting in a total score ranging from 17 to 85. Higher scores indicate higher levels of professional identity. The scale has a KMO value of 0.894, a Cronbach’s alpha of 0.827, and a split-half reliability of 0.842, which demonstrates its good reliability and validity. In this study, the Cronbach’s alpha coefficient for this scale was 0.914.

#### Connor-Davidson resilience scale (CD-RISC)

The Connor-Davidson Resilience Scale (CD-RISC), which was translated by XiaoNan Yu [33], was used in this study. The scale has three dimensions—perseverance, self-reliance, and optimism—and includes a total of 25 items. Respondents are asked to indicate how often they experience each item on a 5-point Likert scale ranging from 0 (“never”) to 4 (“always”). Total scores range from 0 to 100, with higher scores indicating greater psychological resilience. The Cronbach’s alpha coefficient for this scale is 0.910. In this study, the Cronbach’s alpha coefficient for this scale was 0.965.

#### Achievement motivation scale (AMS)

This study used the Achievement Motivation Scale (AMS), which was developed by Norwegian psychologist Gjesme in 1970 and translated into Chinese by Renmin Ye [34]. The scale has two dimensions, motivation for success (Ms) and motivation to avoid failure (Mf), and contains 30 items in total. Respondents are asked to rate each item on a 4-point Likert scale ranging from 1 (“very consistent”) to 4 (“completely inconsistent”). Total scores for both dimensions range from 15 to 60; higher scores indicate stronger achievement motivation. The Cronbach’s alpha coefficient for this scale is 0.867; in this study, it was 0.976.

### Data collection

As researchers, we uploaded the selected scales to Questionnaire Star and created the corresponding questionnaire link. To ensure data quality and prevent omissions or random responses, we set all questions as mandatory and restricted each IP address to a single submission via WeChat. Prior to the survey, we engaged with university officials to thoroughly explain the objectives and significance of the survey, the proper completion methods, and our commitment to anonymity. With their understanding and support, these officials shared the Questionnaire Star link in their respective class WeChat groups for participants to complete. The Questionnaire Star system automatically collected all survey data and exported it in Excel format. A total of 379 questionnaires were collected. After a thorough review by two individuals, 366 valid responses were retained, resulting in an effective response rate of 96.6%.

### Statistical analyses

This study used SPSS 27.0 and Amos 24.0 software for statistical analysis and model construction. Categorical data were presented as frequencies and percentages. Continuous data that were normally distributed were expressed as mean ± standard deviation, independent samples t-tests and one-way ANOVA were used to compare professional identity scores among multiple groups. Pearson correlation analysis was conducted to examine the relationships between the variables. *P* < 0.05 indicated a statistically significant correlation. The Pearson correlation coefficient (r) was interpreted further, with *r* > 0 representing significant positive correlations and *r* < 0 representing significant negative correlations. Stepwise multiple linear regression analysis was performed to assess the effects of creative anxiety, psychological resilience, and achievement motivation on professional identity levels among Master’s Nursing Students. Amos 24.0 was used to construct the chained mediation model, and the bias-corrected percentile bootstrap method was used to test the mediation effects. The PROCESS macro, developed by Hayes, was used to analyze the mediation and moderation effects. Statistical significance of the indirect effects was determined when the 95% bias-corrected confidence intervals did not include zero. The p-value indicates the level of statistical significance. A p-value less than 0.05 suggests that the result is statistically significant.

## Results

### Demographic analysis of master’s nursing students (*n* = 366)

Table 1 summarizes the demographic of the participants. 366 master of nursing students are predominantly female (85.5%) and under the age of 25 (57.7%), with the majority single (93.3%) and in their first year of study (52.5%). In terms of their major, geriatric nursing (23%) was the most common, while community nursing (7.7%) was the least common. In terms of research achievements, almost half of the students (46.7%) had no publications, while 45.6% had one publication. In terms of average weekly self-study time, most students (60.4%) spent ≤ 14 h per week on self-study, and self-reported study efficiency was predominantly moderate (70.8%). For career direction, clinical nursing (45.9%) and nursing education (35.5%) were the main directions. Team communication was mostly moderate to frequent (86.9%), and the majority of students (71.9%) received active supervisory support. The graduation requirement for publication was predominantly in core journals (83.6%), with relatively lower representation in SCI/CSCD (3.3% each).

**Table 1:** General information of master’s nursing students

Variables	Items	*n*	Constituent ratio(%)
Gender	Male Female	53 313	14.5 85.5
Age	<25 25 ~ 30 >30	210 138 18	57.7 37.7 4.9
Marital status	Unmarried Married	342 24	93.3 6.6
Grade	First year master Second year master Third year master	192 111 63	52.5 30.3 17.2
Major	Geriatric nursing Community nursing Acute and critical care Nursing Obstetrics and gynecology Nursing Nursing education Nursing management else	84 28 48 66 36 32 72	23.0 7.7 13.1 18.0 9.8 8.7 19.7
Current research achievements	Nothing One item ≥ 2 items	171 167 28	46.7 45.6 7.7
Average weekly self-study time	<7 h 7–14 h 14–21 h >21 h	109 130 91 36	29.8 35.5 24.9 9.8
Study efficiency	Lower General Higher	73 259 34	19.9 70.8 9.3
Future career direction	Nursing education Clinical care Pursue doctoral studies Else	130 168 29 39	35.5 45.9 7.9 10.7
The level of communication between teams	Few General Frequency	48 180 138	13.1 49.2 37.7
Mentor guidance enthusiasm	Active guidance Little guidance	263 103	71.9 28.1
Master’s degree graduation requirements	Publish general journal articles Publish core journal papers Publish SCI journal papers	36 318 12	9.8 86.8 3.3

### Common method bias test

The Harman single-factor test was used to perform a factor analysis of the items involved in this study. The results indicated that there were 12 factors with eigenvalues greater than 1. The first factor explained 31.24% of the variance, which is below the threshold of 40% [31], indicating that there was no significant common method bias in this study.

### Correlation analysis of creative anxiety professional identity psychological resilience and achievement motivation in master’s nursing students

The results of the correlation analysis for the four variables are shown in Table 2. Creative anxiety was significantly and negatively correlated with professional identity (*r* = − 0.668, *P* < 0.001), psychological resilience (*r* = − 0.537, *P* < 0.001), and achievement motivation (*r* = − 0.503, *P* < 0.001). Conversely, professional identity showed a significant positive correlations with psychological resilience (*r* = 0.731, *P* < 0.001) and achievement motivation (*r* = 0.663, *P* < 0.001). Psychological resilience also showed a significant positive correlations with achievement motivation (*r* = 0.610, *P* < 0.001).

**Table 2:** Correlation analysis of creative anxiety, professional identity, psychological resilience, and achievement motivation in master’s nursing students (*n* = 366)

Item	Creative anxiety	Professional identity	Psychological resilience	Achievement motivation
Creative Anxiety	1.000	-	-	-
Professional Identity	−0.668**	1.000	-	-
Psychological Resilience	−0.537**	0.731**	1.000	-
Achievement Motivation	−0.503**	0.663**	0.610**	1.000
M	12.92	48.47	28.32	6.23
SD	3.58	12.76	7.71	1.83

Note: **P < 0.01.*P < 0.05

### Stepwise multiple linear regression analysis of the factors affecting professional identity of master’s nursing students

A multiple linear regression analysis was performed to examine the impact of creative anxiety, psychological resilience, and achievement motivation on the professional identity of master’s nursing students (Table 3). The results revealed that creative anxiety negatively predicted professional identity (*P* < 0.001), whereas psychological resilience and achievement motivation positively predicted it (*P* < 0.001). Together, these three variables explained 66.6% of the total variance in professional identity. Additionally, variance inflation factors (VIF) were all less than 5, indicating no multicollinearity among the variables.

**Table 3:** Multiple linear regression analysis of the factors affecting professional identity of master’s nursing students

Variable	B	β	t	*R* ^2^	*P*	VIF
**Level 1** (Constant)	24.788		42.784	44.7	<0.001	
Creative Anxiety	−1.481	−0.668	−17.147		<0.001	1.000
**Level 2** (Constant)	13.700		14.031	62.3	<0.001	
Creative Anxiety	−0.888	−0.401	−10.492		<0.001	1.406
Psychological Resilience	0.769	0.489	13.031		<0.001	1.406
**Level 3** (Constant)	10.992		10.951	66.6	<0.001	
Creative Anxiety	−0.742	−0.335	−8.961		<0.001	1.509
Psychological Resilience	0.570	0.369	9.062		<0.001	1.796
Achievement Motivation	0.583	0.370	6.784		<0.001	1.709

### Mediation effect of psychological resilience and achievement motivation in the relationship between creative anxiety and professional identity

This study constructed a chain mediation model using Amos 24.0 based on relevant literature and statistical analysis. The model included creative anxiety as the independent variable, psychological resilience and achievement motivation as mediators, and professional identity as the dependent variable. The results indicated a good model fit [CMIN/DF = 1.228(<5), GFI = 0.975(>0.90), RMR = 0.027(<0.05), AGFI = 0.960(>0.90), and RMSEA = 0.025(<0.05)].

**Fig. 2:**
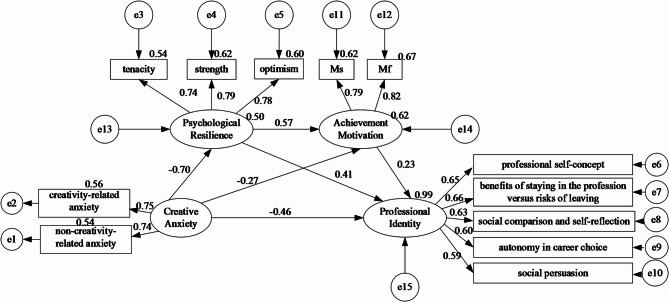
Chain mediation model of psychological resilience and achievement motivation in the relationship between

Figure 2 and the accompanying Table 4 delineated the outcomes of the mediation model. The results showed that creative anxiety has an impact on both the direct and indirect effects of professional identity. The direct effect was − 0.124, and the total indirect effect was − 0.334. All specific and chain mediation effects were also significant.

**Creative anxiety**→**Professional identity:** Creative anxiety can directly affect the professional identity of master’s degree nursing students. The effect value was − 0.124, accounting for 53.32% of the total effect. Creative anxiety had a negative impact on professional identity.

**Creative anxiety**→**Psychological resilience**→**Professional identity:** Psychological resilience mediates the relationship between creative anxiety and professional identity in master’s nursing students. The mediation effect value was − 0.70 × 0.41 = − 0.287, accounting for 24.21% of the total indirect effect. The negative impact of creative anxiety on professional identity was partially mitigated through the mediating role of psychological resilience.

**Creative anxiety**→**Achievement motivation**→**Professional identity:** Achievement motivation mediates the relationship between creative anxiety and professional identity in master’s nursing students. The mediation effect value was − 0.27 × 0.23 = − 0.062, accounting for 13.50% of the total indirect effect. Achievement motivation attenuated the adverse effect of creative anxiety on professional identity by serving as a mediator.

**Creative anxiety**→**Psychological resilience**→**Achievement motivation**→**Professional identity:** Both psychological resilience and achievement motivation mediate the relationship between creative anxiety and professional identity in master’s nursing students. The mediation effect value was − 0.70 × 0.57 × 0.23 = − 0.092, accounting for 8.97% of the total indirect effect. Psychological resilience indirectly enhanced professional identity by increasing achievement motivation, thereby reducing the detrimental influence of creative anxiety.

**Table 4:** Mediation effect analysis (Standardized)

Path	β	Boot SE	Bootstrap 95%CI	Percent (%)
Total effect	−0.458	0.052	−0.531 ~ − 0.230	-
Direct effect(Creative anxiety→Professional identity)	−0.124	0.014	−0.151 ~ − 0.096	53.32
Indirect effect	−0.334	0.032	−0.397 ~ − 0.270	46.68
Creative anxiety→Psychological resilience→Professional identity	−0.287	0.072	−0.437 ~ − 0.152	24.21
Creative anxiety→Achievement motivation→Professional identity	−0.062	0.033	−0.150 ~ − 0.012	13.50
Creative anxiety→Psychological resilience→Achievement motivation→Professional identity	−0.092	0.049	−0.211 ~ − 0.009	8.97

## Discussion

This study investigated the impact of creative anxiety on professional identity among master’s nursing students, examining psychological resilience and achievement motivation as mediators. Key findings confirm that creative anxiety directly reduces professional identity and also exerts indirect effects through both the independent mediating roles and a chain-mediating pathway involving psychological resilience and achievement motivation. The findings provide a crucial theoretical foundation for optimizing psychological interventions in advanced nursing education and hold significant practical value for enhancing professional identity and reducing talent attrition within the profession.

The descriptive findings indicated that the total score of creative anxiety among master’s nursing students was 12.92 ± 3.58, which was higher than the scores reported in undergraduate students [31]. The master’s nursing students who, in addition to developing clinical competencies, were required to undertake tasks demanding creative thinking such as research exploration. Simultaneously, they faced challenges including high academic expectations and inadequate supervision [5]; these factors collectively contributed to the observed higher creative anxiety. Conversely, the total professional identity score was 48.47 ± 12.76, lower than the findings reported by Liao et al. [35]. The low score may be attributed to contextual factors: First, the combined effect of academic pressure and creative anxiety may undermine their professional confidence, while the conflict between high-intensity research demands and clinical practice can easily lead to a blurred sense of professional value [36]. Second, the traditional societal perception of nursing as a discipline creates a gap with graduate students’ high expectations for the profession, and the marginalization of nursing roles in the healthcare environment further exacerbates the identity crisis [37].

### Direct effect of creative anxiety on professional identity

This study found a significant direct negative influence of creative anxiety on the professional identity of master’s nursing students, with a mediation effect value of − 0.124, accounting for 53.32% of the total effect. Master’s nursing students often experience a challenging transition from clinical practice to academic roles [38]. This liminal phase, characterized by uncertainty and perceived or actual threats, frequently culminates in anxiety [39]. Within research and clinical innovation contexts, creative anxiety functions as a threatening stressor. Without adequate support systems, these combined stressors increase the risk of early-career mental health issues, thereby impeding the development of a stable professional identity [40].

According to the COR framework, creative anxiety, viewed as a threat to psychological resources, depletes an individual’s internal resources (such as cognitive and emotional energy) and may lead to resource loss [13]. The emotional exhaustion (e.g., frustration, self-doubt) could cause master’s nursing students to detach from their work, thereby negatively impacting their professional identity [41]. Research by Daker et al. [17]also indirectly suggests that creative anxiety may trigger negative professional emotional experiences, weaken career motivation, and consequently impair professional identity. Therefore, this finding implies that interventions aimed at alleviating creative anxiety among nursing master’s students could enhance their professional identity.

### The mediating effect of psychological resilience

The analysis further identified psychological resilience as a significant mediator in the relationship between creative anxiety and professional identity among master’s nursing students, with a mediation effect value of − 0.287, accounting for 24.21% of the total indirect effect. This role aligns with previous research indicating that psychological resilience serves as a reliable mediator of professional identity among Chinese nursing students [42]. As a core resource enabling individuals to maintain adaptation and growth under stress [43], psychological resilience can buffer resource threats (e.g., creative anxiety) and promote resource recovery, thus is considered a protective resource [44]. A recent study has shown that psychological resilience is intrinsically linked to creativity through its neurobiological underpinnings, which may serve as a central mediating mechanism against creative anxiety [45]. Based on COR theory, individuals actively invest resources to prevent stressful loss cycles of resources and to enhance motivating resource gain spirals. Therefore, when individuals face creative anxiety (a resource threat), they can achieve resource accumulation (enhanced professional identity) by leveraging psychological resilience (the protective resource). When confronted with creative anxiety, highly resilient individuals may maintain emotional stability, avoid prolonged emotional exhaustion, and prevent anxiety from spreading to the domain of professional cognition [46]. This finding is consistent with similar research demonstrating the mediating effect of psychological resilience in the relationship between nurses’ self-efficacy and professional identity [24]. Consequently, psychological resilience emerges as a strategy to mitigate the impact of creative anxiety, thereby improving professional identity.

### The mediating effect of achievement motivation

Achievement motivation was found to mediate the relationship between creative anxiety and professional identity among master’s nursing students, with a mediation effect value of − 0.062, accounting for 13.50% of the total indirect effect, which aligns with the prior investigations in nursing intern students [15]. As an intrinsic drive for success and excellence, achievement motivation plays a positive role in the academic journey of nursing master’s students. A positive correlation between achievement motivation and professional identity has been well established. From the COR perspective, achievement motivation functions as a “motivational resource” that facilitates the acquisition of resources (e.g., professional identity). Previous study showed decreased professional identity reflecting their stress response [28]. Individuals with high achievement motivation are more likely to adopt effective coping strategies in anxiety-provoking situations, thereby enhancing their sense of professional identity [47].

### Chain mediation effects of psychological resilience and achievement motivation

The findings of this study reveal a chain-mediating role of psychological resilience and achievement motivation between creative anxiety and professional identity among master’s nursing students, with a mediation effect value of − 0.092, accounting for 8.97% of the total indirect effect. The identified pathway is “creative anxiety → psychological resilience → achievement motivation → professional identity”. Relevant studies indicate that individuals with high psychological resilience cognitively reframe anxiety into incentive-based challenges [48], maintaining emotional stability while accumulating achievement motivation—a process that strengthens professional identity development [49]. As posited by the COR theory, psychological resilience, as a protective resource, buffers the depletion of psychological energy caused by creative anxiety, thereby enabling the acquisition and protection of new resources like achievement motivation. In this chain, achievement motivation serves as a critical bridge connecting individual capability (psychological resilience) with career development outcomes (professional identity), which was consistent with that of Luo et al. [26]. Individuals with strong psychological resilience can maintain a positive mindset under pressure, which in turn activates their achievement motivation [50]. Consequently, they gain a sense of fulfillment, enabling them to actively complete work and academic tasks. This dynamic process reframes creative anxiety-provoking stimuli during their studies and enhances their professional identity. This suggests that nursing educators can alleviate creative anxiety and enhance professional identity by strengthening students’ psychological resilience and achievement motivation, thereby providing a foundation for developing scientifically sound and effective interventions. Future research could focus on constructing a tiered psychological resilience training system (e.g., cognitive restructuring training [51], building resilience interventions [52], mindfulness-based psychological interventions [53]), enhancing achievement motivation-driven effects (e.g., phased incentive mechanisms [54], positive motivation education [55]), and implementing digital interventions and resource integration (e.g., AI-powered personalized resilience training platforms, cross-institutional resilience resource sharing [56]).

## Restriction

Limitations of this study: The instruments used in this study relied on self-reported outcomes, which can introduce subjectivity into the results and potentially lead to bias in the collected data, thereby limiting the generalizability of the findings. Future research could address these limitations by incorporating physiological indicators (e.g., cortisol levels) to objectively measure anxiety states. Longitudinal study designs with repeated measurements at different academic stages (e.g., pre- and post-proposal defense) could help control for transient emotional fluctuations. Additionally, qualitative interviews with multiple stakeholders, such as nursing graduate students, faculty advisors, and clinical preceptors, could validate the accuracy of the self-reported data. Furthermore, this study only included 366 master’s nursing students from four universities in Shaanxi Province, which is a relatively small and nonrepresentative sample. Consequently, the results may not be generalizable to populations in other geographical regions. Future studies should expand the sample size and include master’s nursing students from other provinces to enhance the external validity of the findings. Third, as a cross-sectional study, our research may not account for other potential influencing factors. The underlying mechanisms and possible additional mediating effects require further investigation. Future studies could employ longitudinal designs to examine dynamic relationships between variables and conduct large-scale, multicenter surveys to verify the universality of conclusions.

## Conclusion

This study examined 366 master’s nursing students to validate the independent and chain mediating effects of psychological resilience and achievement motivation on the relationship between creative anxiety and professional identity. The results also revealed a negative correlation between creative anxiety and professional identity. To better understand the mechanisms underlying this relationship, the study provides theoretical support for implementing comprehensive interventions to alleviate creative anxiety. The research highlights that psychological resilience and achievement motivation are key mediators of this relationship for master’s nursing students. The findings suggest that managers should address creative anxiety among these students and promote psychological resilience and achievement motivation to strengthen their professional identity.

## Data Availability

Due to the privacy of the study participants, the data generated in the course of this study will not be made public, but can be made available to the corresponding author upon reasonable request.

## Abbreviations

CAS: Creativity Anxiety Scale
PISNS: Professional Identity Scale for Nursing Students
CD-RISC: Connor-Davidson Resilience Scale
AMS: The Achievement Motivation Scale
Ms: Motivation to pursue success
Mf: Motivation to avoid failure
CMIN/DF: Discrepancy Chi Square/degree of freedom
GFI: Goodness-of-fit index
RMR: Root mean square residual
M: Mean
AGFI: Adjusted Goodness of Fit
RMSEA: Root-mean-square error of approximation

## References

[1] McClunie-Trust P, Jarden R, Marriott P, Winnington R, Dewar J, Shannon K, Jones S, Jones V, Turner R, Cochrane L, et al. Graduate entry nursing students’ development of professional nursing self: a scoping review. Int J Nurs Stud. 2024;151:104670. 38215688 10.1016/j.ijnurstu.2023.104670

[2] Ammari N, Gantare A. The impact of university-based education on nursing professional identity: a qualitative examination of students’ experiences. Int J Nurs Educ Scholarsh. 2023;20(1). 10.1515/ijnes-2022-0086 37352478

[3] Zhang C, Xu C, Wang R, Han X, Yang G, Liu Y. The learning experiences and career development expectations of Chinese nursing master’s degree students: A qualitative investigation. Nurse Educ Pract. 2024;77:103996. 38763114 10.1016/j.nepr.2024.103996

[4] Homayouni L, Zare A, Padam Z, Fereidouni A. Investigating academic dishonesty and its relationship with moral competence and professional identity of nursing students: a cross-sectional study. BMC Nurs. 2024;23(1):662. 39289688 10.1186/s12912-024-02335-8 PMC11409767

[5] Strout K, Schwartz-Mette R, McNamara J, Parsons K, Walsh D, Bonnet J, O’Brien LM, Robinson K, Sibley S, Smith A, et al. Wellness in nursing education to promote resilience and reduce burnout: protocol for a holistic multidimensional wellness intervention and longitudinal research study design in nursing education. JMIR Res Protoc. 2023;12:e49020. 37682598 10.2196/49020 PMC10517386

[6] Kim G, Yu H, Ryu E. Social group membership, burnout, and subjective well-being in new nurses in the life transition period: A cross-sectional study. Nurs Open. 2023;10(5):3295–304. 36575584 10.1002/nop2.1581 PMC10077367

[7] Boamah SA, Read EA, Spence Laschinger HK. Factors influencing new graduate nurse burnout development, job satisfaction and patient care quality: a time-lagged study. J Adv Nurs. 2017;73(5):1182–95. 27878844 10.1111/jan.13215

[8] Browne C, Wall P, Batt S, Bennett R. Understanding perceptions of nursing professional identity in students entering an Australian undergraduate nursing degree. Nurse Educ Pract. 2018;32:90–6. 30098517 10.1016/j.nepr.2018.07.006

[9] Zhao X, Zheng WK, Wang XH, Fang J, Chen WJ, Li N, Wen HT, Feng XJ, Wang MF, Heng CN, et al. Influence of perceived stress on professional identity among nursing students: a chain mediating role of self-control and self-directed learning ability. Front Med (Lausanne). 2024;11:1429014. 39600932 10.3389/fmed.2024.1429014 PMC11588480

[10] Mei XX, Wang HY, Wu XN, Wu JY, Lu YZ, Ye ZJ. Self-efficacy and professional identity among freshmen nursing students: a latent profile and moderated mediation analysis. Front Psychol. 2022;13:779986. 35310284 10.3389/fpsyg.2022.779986 PMC8927723

[11] Demerouti E. Job demands-resources and conservation of resources theories: how do they help to explain employee well-being and future job design? J Bus Res. 2025;192.

[12] Hobfoll SE. The influence of culture, community, and the nested-self in the stress process: advancing conservation of resources theory. Appl Psychol. 2001;50(3).

[13] Prapanjaroensin A, Patrician PA, Vance DE. Conservation of resources theory in nurse burnout and patient safety. J Adv Nurs. 2017;73(11):2558–65. 28543427 10.1111/jan.13348

[14] Fitzgerald A. Professional identity: A concept analysis. Nurs Forum. 2020;55(3):447–72. 32249453 10.1111/nuf.12450

[15] Zhu J, Xie X, Pu L, Zou L, Yuan S, Wei L, Zhang F. Relationships between professional identity, motivation, and innovative ability among nursing intern students: A cross-sectional study. Heliyon. 2024;10(7):e28515. 38596131 10.1016/j.heliyon.2024.e28515 PMC11002581

[16] Daker RJ, Cortes RA, Lyons IM, Green AE. Creativity anxiety: evidence for anxiety that is specific to creative thinking, from STEM to the arts. J Exp Psychol Gen. 2020;149(1):42–57. 31219299 10.1037/xge0000630

[17] Daker RJ, Viskontas IV, Porter GF, Colaizzi GA, Lyons IM, Green AE. Investigating links between creativity anxiety, creative performance, and state-level anxiety and effort during creative thinking. Sci Rep. 2023;13(1):17095. 37816728 10.1038/s41598-023-39188-1 PMC10564955

[18] Tehranineshat B, Rakhshan M. The relationship between knowledge management and creativity in bachelor degree compared to master degree nursing students. Invest Educ Enferm. 2018;36(3). 10.17533/udea.iee.v36n3e05 31083851

[19] Gonzalez-Dolginko B, Netzer D. Creative expression as transitional object: bridging Personal-Professional identity. Psychoanal Rev. 2021;108(1):79–96. 33617337 10.1521/prev.2021.108.1.79

[20] Arreciado Marañón A, Isla Pera MP. Theory and practice in the construction of professional identity in nursing students: a qualitative study. Nurse Educ Today. 2015;35(7):859–63. 25863650 10.1016/j.nedt.2015.03.014

[21] Du J, Wu C, Zheng WK, Cui SN, Li L, Liu Z, Gao L, Heng CN, Lang HJ. Insomnia and anxiety among COVID-19 patients in china: the chain mediating effect of psychological capital and self-esteem. BMC Nurs. 2024;23(1):221. 38561710 10.1186/s12912-023-01563-8 PMC10983642

[22] Huang Houling. A study on the relationship between achievement motivation, mental health, and creativity tendency among master of nursing students. Master’s Thesis. 2018.

[23] Zhang L, Zhang Q, Li S, Li Y, Wu G, Chen Y, Zhou Y. The mediating role of psychological resilience in Chinese nursing students’ professional identity and learning burnout. J Korean Acad Nurs. 2024;54(4):509–18. 39663616 10.4040/jkan.24044

[24] Yi R, Zhou Z, Ma W, Yang C, Wang F, Wu J. Mediating role of psychological resilience in the relationship between self-efficacy and professional identity among nurses. Biotechnol Genet Eng Rev. 2024;40(2):726–38. 36966473 10.1080/02648725.2023.2190943

[25] McCoy TP, Hoffart N, Lewallen LP, Thorpe S. The validity and reliability of professional Self-Concept in nursing in accelerated bachelor’s and master’s nursing students. J Nurs Meas. 2020;28(2):382–403. 32430356 10.1891/JNM-D-18-00107

[26] Luo J, Liu XB, Yao Q, Qu Y, Yang J, Lin K, Pan SR, Wang TY, Dai Y, Chen HY, et al. The relationship between social support and professional identity of health professional students from a two-way social support theory perspective: chain mediating effects of achievement motivation and meaning in life. BMC Med Educ. 2024;24(1):473. 38685015 10.1186/s12909-024-05391-5 PMC11059822

[27] Bon AT, Shire AM. Review of conservation of resources theory in job demands and resources model. Int J Global Optim its Application. 2022;1(4):236–48.

[28] Peng Y, Zhang C. The influence of achievement motivation on the educational practice ability of pre-service teachers: the multiple mediating role of professional identity and learning engagement. Front Psychol. 2024;15:1463557. 39372963 10.3389/fpsyg.2024.1463557 PMC11449695

[29] Li J, Pan X. The impact of mood on sports flow state in football players: a chain mediating model of psychological resilience and achievement motivation in competition. Front Psychol. 2025;16:1523400. 40417032 10.3389/fpsyg.2025.1523400 PMC12098437

[30] Fonseca M. Principles and practice of structural equation modeling, third edition by Rex B. Kline. Int Stat Rev. 2013;81(1):172–3.

[31] Ren Zhiting. Measurement of creative anxiety and its neural basis in brain function. Master’s Thesis. 2020.

[32] Hao Yufang. A study on the self-education model for enhancing nursing students’ professional identity and occupational self-efficacy. Ph.D. Dissertation. 2011.

[33] Yu Xiaonan, Zhang Jianxin. A comparative study on the application of the self-reliability scale and the Connor-Davidson resilience scale. J Psycho Sci. 2007(05):1169–71.

[34] Ye Renmin KH. Measurement and analysis of achievement motivation. Psycho Dev Edu. 1992(02):14–6.

[35] Liao M, Xie Z, Ou Q, Yang L, Zou L. Self-efficacy mediates the effect of professional identity on learning engagement for nursing students in higher vocational colleges: A cross-sectional study. Nurse Educ Today. 2024;139:106225. 38718534 10.1016/j.nedt.2024.106225

[36] Abbate S, Gambino KM, Timoney PM, Wellins AM, Savoca M, Zalewski U. Identifying professional goals and promoting career development in nursing students through reflection and simulation. Nurs Educ Perspect; 2024. 10.1097/01.NEP.0000000000001329 39287647

[37] Crossen EM, Hunter Revell SM. Feeling valued as a Mid-Career nurse: A scoping review. J Nurses Prof Dev. 2024;40(5):242–7. 39103980 10.1097/NND.0000000000001078

[38] Vo TN, Chiu HY, Chuang YH, Huang HC. Prevalence of stress and anxiety among nursing students: A systematic review and Meta-analysis. Nurse Educ. 2023;48(3):E90–5. 36538669 10.1097/NNE.0000000000001343

[39] Chi T, Cheng L, Zhang Z. Global prevalence and trend of anxiety among graduate students: A systematic review and meta-analysis. Brain Behav. 2023;13(4):e2909. 36852520 10.1002/brb3.2909 PMC10097092

[40] Yiu S, Yeung M, Cheung WJ, Frank JR. Stress and conflict from Tacit culture Forges professional identity in newly graduated independent physicians. Adv Health Sci Educ Theory Pract. 2023;28(3):847–70. 36477578 10.1007/s10459-022-10173-z

[41] Maslach C, Leiter MP. Understanding the burnout experience: recent research and its implications for psychiatry. World Psychiatry. 2016;15(2):103–11. 27265691 10.1002/wps.20311 PMC4911781

[42] Sang N, Zhu ZZ, Wu L, Shi PL, Wang LW, Kan HY, Wu GC. The mediating effect of psychological resilience on empathy and professional identity of Chinese nursing students: A structural equation model analysis. J Prof Nurs. 2022;43:53–60. 36496245 10.1016/j.profnurs.2022.09.002

[43] McKinzie C, Altamura V, Burgoon E, Bishop C. Exploring the effect of stress on mood, self-esteem, and daily habits with psychology graduate students. Psychol Rep. 2006;99(2):439–48. 17153812 10.2466/pr0.99.2.439-448

[44] Troy AS, Willroth EC, Shallcross AJ, Giuliani NR, Gross JJ, Mauss IB. Psychological resilience: an Affect-Regulation framework. Annu Rev Psychol. 2023;74:547–76. 36103999 10.1146/annurev-psych-020122-041854 PMC12009612

[45] Ren Z, Yang W, Qiu J. Neural and genetic mechanisms of creative potential. Curr Opin Behav Sci. 2019;27:40–6.

[46] Yang JS, Jeon YJ, Lee GB, Kim HC, Jung SJ. The association between psychological resilience and cognitive function in longitudinal data: results from the community follow-up survey. J Affect Disord. 2021;290:109–16. 33993077 10.1016/j.jad.2021.04.062

[47] Ye T, Luo J, Chen Y, Huang Y, He M, Yang J, Wang T, Yao Q, Qu Y, Yang Z. The influence of meaning in life on smartphone addiction among nursing undergraduates: the mediating roles of professional identity and achievement motivation. BMC Nurs. 2025;24(1):258. 40055711 10.1186/s12912-025-02781-y PMC11887170

[48] Zhang Y, Liu F, Ma J, Wu J, Shen C, Chang F, Hu W, Lang H. Psychological stress and depression symptoms in nursing undergraduates: the chain mediating effect of cognitive reappraisal and ruminate thinking. BMC Nurs. 2025;24(1):8. 39762876 10.1186/s12912-024-02604-6 PMC11702140

[49] Cook DA, Castillo RM, Gas B, Artino AR Jr. Measuring achievement goal motivation, mindsets and cognitive load: validation of three instruments’ scores. Med Educ. 2017;51(10):1061–74. 28901645 10.1111/medu.13405

[50] Beauvais AM, Stewart JG, DeNisco S, Beauvais JE. Factors related to academic success among nursing students: a descriptive correlational research study. Nurse Educ Today. 2014;34(6):918–23. 24380623 10.1016/j.nedt.2013.12.005

[51] Morello K, Schäfer SK, Kunzler AM, Priesterroth LS, Tüscher O, Kubiak T. Cognitive reappraisal in mHealth interventions to foster mental health in adults: a systematic review and meta-analysis. Front Digit Health. 2023;5:1253390. 37927578 10.3389/fdgth.2023.1253390 PMC10623449

[52] Yu F, Chu G, Yeh T, Fernandez R. Effects of interventions to promote resilience in nurses: A systematic review. Int J Nurs Stud. 2024;157:104825. 38901125 10.1016/j.ijnurstu.2024.104825

[53] Sulosaari V, Unal E, Cinar FI. The effectiveness of mindfulness-based interventions on the psychological well-being of nurses: A systematic review. Appl Nurs Res. 2022;64:151565. 35307128 10.1016/j.apnr.2022.151565

[54] Howard JL, Bureau J, Guay F, Chong JXY, Ryan RM. Student motivation and associated outcomes: A Meta-Analysis from Self-Determination theory. Perspect Psychol Sci. 2021;16(6):1300–23. 33593153 10.1177/1745691620966789

[55] Henrique-Sanches BC, Cecilio-Fernandes D, Costa RRO, Almeida R, Etchegoyen FF, Mazzo A. Implications of clinical simulation in motivation for learning: scoping review. Einstein (Sao Paulo). 2024;22:Rw0792. 38695476 10.31744/einstein_journal/2024RW0792 PMC11081016

[56] Zhao W, Chen S, Hu J, Zhou Q, Tao J, Gao R, Zhang J, Su S, Wang Y, Su Y, et al. The applicability and efficacy of Micro-Video psychological training camp in groups with mild to moderate symptoms of depression and anxiety: A prospective and randomized controlled trial protocol. Front Psychiatry. 2022;13:991465. 36733416 10.3389/fpsyt.2022.991465 PMC9887015

